# Barriers and opportunities for return-to-work of cancer survivors: time for action—rapid review and expert consultation

**DOI:** 10.1186/s13643-016-0210-z

**Published:** 2016-02-24

**Authors:** Régine Kiasuwa Mbengi, Renée Otter, Katrien Mortelmans, Marc Arbyn, Herman Van Oyen, Catherine Bouland, Christophe de Brouwer

**Affiliations:** Center for Environmental Health and Occupational Health, School of Public Health, Université Libre de Bruxelles, Brussels, Belgium; Belgian Cancer Center, Department of Public Health and Surveillance, Scientific Institute of Public Health, Brussels, Belgium; Unit of Cancer Epidemiology, Department of Public Health and Surveillance, Scientific Institute of Public Health, Brussels, Belgium; Department of Public Health and Surveillance, Scientific Institute of Public Health, Brussels, Belgium; Department of Research and Development, Mensura, Antwerp, Belgium

**Keywords:** Return-to-work, Rapid review, Risk factors, Cancer survivors, Expert consultation

## Abstract

**Background:**

The spread of early detection and the improvement of cancer treatment have led to an increased prevalence of cancer survivors, including in the working age population. Return-to-work (RTW) of cancer survivors has become a key issue for national cancer control plans. This study aims (1) to identify the factors that have an impact on RTW of cancer survivors and to draw a risk profile supporting health professionals in the screening of those at risk for barriers of RTW and (2) to sharpen these results with input from health, social security and academic Belgian experts and to provide evidence-based recommendations that facilitate RTW of cancer survivors.

**Methods:**

A rapid review was conducted, based on the methodology elaborated by The Knowledge to Action Research Programme and researchers from the University of York, including a quality assessment of retained studies. Next, the Delphi method was used to organize a consultation with experts in order to discuss, validate and complement the results.

**Results:**

Forty-three out of 1860 studies were included. We identified nine risk factors grouped into four categories: socio-demographic, disease and treatment-related, work-related, and personal and subjective factors. Experts suggested dividing them into two even groups: factors which are modifiable and those which are not. The awareness of health professionals regarding the identified factors, a better assessment of work capacities, clarity on the rights and obligations of employers and workers alike, and the setup of a positive discrimination employment policy for cancer survivors were acknowledged as factors facilitating RTW of cancer survivors.

**Conclusions:**

The awareness of health professionals regarding barriers of RTW may improve the early identification of cancer survivors at risk for prolonged time to RTW and may allow early supportive intervention. Social and employment policies should be better tailored to support both employers and cancer survivors in the RTW process, providing incentives to positively discriminate cancer survivors on prolonged sick leave.

**Electronic supplementary material:**

The online version of this article (doi:10.1186/s13643-016-0210-z) contains supplementary material, which is available to authorized users.

## Background

Over the last decades, progress in early cancer detection and treatment has led to a significant decrease of cancer mortality rates in developed countries [1]. A lower mortality combined with higher incidence has led to a higher prevalence of cancer survivors [2]. In 2012, the 5-year prevalence of cancer in Belgium numbered over 192,000 cases.^1^

**Fig. 1:**
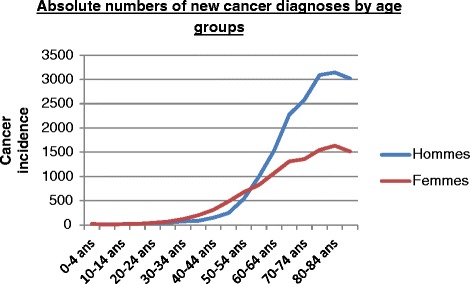
Age-specific incidence rate in males and females in Belgium, 2011 (*n*/100,000 person years). Belgian Cancer Registry

Despite the progress in treatment, cancer survivors have to live with adverse effects of treatment over the medium to long term. These effects—whether physical or emotional—can negatively affect all aspects of their lives, including their capacity to maintain a professional activity [3]. In 2011, a total of 69,062 new cancer diagnoses were registered in Belgium, including 27,316 (40 %) in the working age population (Fig. 1).

The ability to remain (even partially) professionally active is an important added value to maintain a good quality of life, from an economic and social perspective [4]. In Belgium, each year, sickness absence and disability cost over 2 % of the OECD gross product [5]. Given the increasing number of cancer survivors, their ongoing participation in work may add to the sustainability of social security systems.

The reintegration of cancer survivors into society needs to be one of the main goals of National Cancer Control Plans (NCCPs). At the moment, no such initiative exists in the Belgian NCCP. This has led the health, social security and employment policy decision-makers to call for evidence-based recommendations, in order to address this gap [6].

First, this study aims to identify the factors that have an impact on return-to-work (RTW) of cancer survivors, and to draw a risk profile, that supports health professionals in the screening of those at risk to encounter difficulties to resume work. The second aim of the study is to sharpen these results with input from experts on health, employment and social security, in order to provide recommendations on RTW of cancer survivors.

## Methods

### Selection of studies

**Fig. 2:**
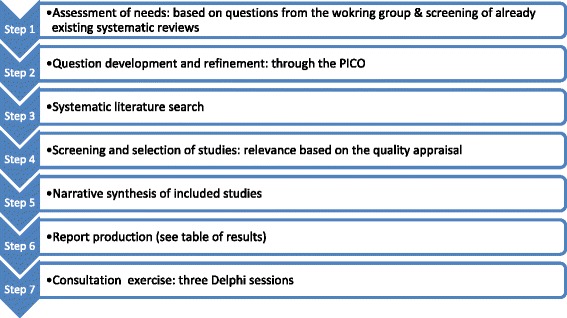
The stepwise approach followed to conduct the rapid review. Adapted from Arksey and O’Malley [8] and Khangura et al. [7]

We conducted this rapid review, based on the methodology elaborated by The Knowledge to Action Research Programme [7] and by researchers from the University of York [8]. The seven steps followed to complete this study are presented in Fig. 2. This rapid review was not registered with PROSPERO, and no ethical approval or informed consent was required.

To optimize the search, we framed a PICO. The population (P) is *working age cancer patients* (*18–64*), the indicators (I) are all those related to *the employment of cancer patients*, the comparison (C) is done among patients with different cancer sites or affected by other diseases or disease-free adults, and the outcome (O) of interest is *RTW* or *employment*. We limited our search to studies published in the period January 2000 until January 2015 We excluded studies involving patients with a poor prognosis (i.e. survival less than 12 months) and focused on studies that primarily investigated the possible effects of cancer on RTW and employment status.

**Fig. 3:**
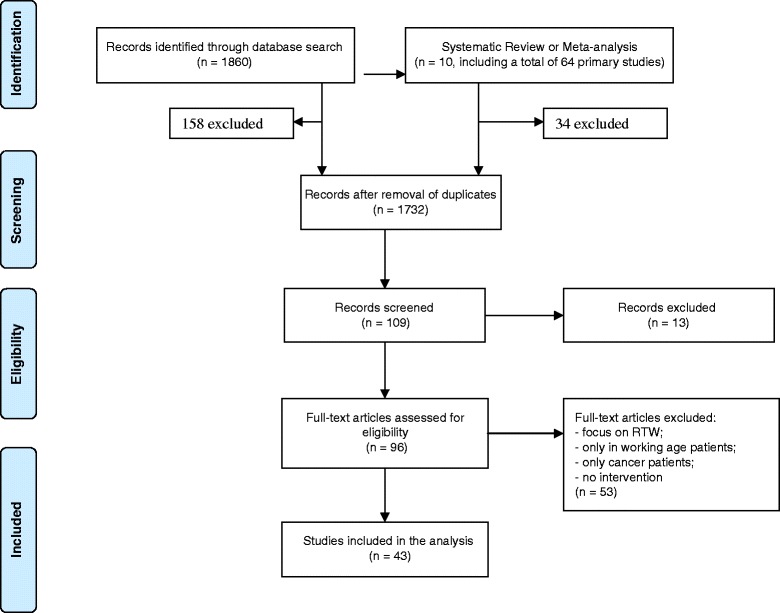
The PRISMA flow chart of included studies

We searched through three databases: MEDLINE, Cochrane Library and Trip Database. The following MeSH and non-MeSH terms were used to perform the search: *rehabilitation*, *employment status*, *survivorship*, *survivors*, *long-term survivors*, *cancer patients*, *return-to-work*. In total, the primary search delivered 1860 papers as presented in the PRISMA flow chart of Fig. 3.

We first focused on systematic reviews and meta-analyses. The search resulted into 10 systematic reviews, three of which fitted our research questions: de Boer et al. 2009, van Muijen et al. 2013 and Banning et al. 2011 [9–11]. Out of the 64 original studies included in these three systematic reviews, we picked up 30 of them, after having eliminated the duplications and those that did not meet our specific criteria. Second, we compared the results from the additional search of original studies and added 13 studies, ending with a total of 43 original studies.

### Quality assessment

Since we included all types of studies, we decided to assess their methodological quality and level of evidence, in order to value their relevance.

To define the criteria for the quality assessment, we decided to merge three validated assessment tools: the checklist of Downs and Black, the MINORS instrument and the CASP checklist for qualitative research [12–14]. The three tools delivered 49 questions that we grouped into three categories: questions related to internal validity, external validity and other questions. All questions were screened and compared, and those which recurred in all three tools were kept to fit our list of criteria that we used in our quality assessment. As presented in Table 3, 10 criteria have been retained, nine of which counting for a maximum of three points and one for two points. Each study could get a maximum score of 29 points, and we divided the studies into three groups: studies scoring 24 points or over (i.e. meeting most validity criteria), those scoring between 18 and 23 points (i.e. meeting over half of the validity criteria) and studies with a score of 17 points or less, meaning poor validity (Table 1).

**Table 1:** Criteria to assess the relevance of studies included in the analysis

Categories	Criteria	Points
Internal validity	Generalities	
	Is the hypothesis/aim/objective of the study clearly described?	3
	Are the main findings/results/outcomes clearly described?	3
	Are the characteristics of the patients included described?	(3)
	Socio-demographic	1
	Disease/treatment-related	1
	Work-related	1
	Study design and methods	
	Data collection	
	Population-based	3
	Regionally based (or several settings)	2
	Hospital/centre-based	1
	Were the groups equivalent at baseline (or adjusted)?	(3)
	Socio-demographic characteristics	1.5
	Work-related characteristics	1.5
	Number subjects included	
	≥500	3
	≥50, <500	2
	<50	1
	Length of follow-up (since diagnosis or treatment)	
	≥1 year	3
	≥6 months	2
	≥3 months	1
	Loss of follow-up = less than 5 %	2
External validity	Number of cancer sites	
	>10	3
	4–10	2
	≥3	1
	Were the subjects representative of the population from which they were recruited?	3
Total (maximum)		29

The quality assessment gave the following results: 10 studies scored a ‘high relevance’, 19 ‘a moderate relevance’ and 14 ‘a low relevance’ (Table 3).

### Consultation with experts

In order to validate the factors identified through the literature review, we used the Delphi method [15] and presented the results to a group of experts. We invited participants from different fields of expertise: academics, government officials, health professionals, social carers and patients’ representatives (Table 2). The researchers contacted the main Belgian institutions (head of relevant departments) involved in the professional reintegration after sickness, explaining the objectives of the study. Next, the participating experts were appointed by their institution. The objective of this consultation was to ensure the relevance, in the Belgian context, of the risk factors identified in literature and also to assess their relevance related to the ability of cancer survivors to remain occupationally active.

**Table 2:** List of experts having participated in the Delphi sessions

Participants	Institution	Position
	Governmental	
	National Institute for Health and Disability Insurance (NIHDI)	Director
2	NIHDI	Civil servant (officer)
	Federal Public Service (Ministry) Public Health	Civil servant (officer)
2	Federal Public Service (Ministry) Employment	Civil servant (officer)
	Sickness funds	
	Christelijke Mutualiteit (CM)	Medical advisor for the CM
	University hospitals	
2	UZ Gent	Psycho-oncologist
	Cliniques universitaires Saint Luc	Psychologist
	Cliniques universitaires Saint Luc	Coordinator of oncological care
	Cliniques universitaires Saint Luc	Psycho-oncologist
	Institut Jules Bordet	Coordinator of oncological care (nurse)
	Institut Jules Bordet	Occupational therapist
	Institut Jules Bordet	Oncologist
	Hôpital Universitaire Erasme (ULB)	Physiotherapist
	KU Leuven, Occupational, Environmental and Insurance Medicine	Researcher, PhD
	KU Leuven, Institute of Labour Law	Research assistant, PhD
	ULg, Occupational Health and Health Education	Professor of occupational medicine
	ULg, Occupational Health and Health Education	PhD student, occupational health
	HELB, Occupational Therapy	Occupational therapist, professor
	General hospital	
	CHC de Liège	Oncologist
	Patient’s associations and foundations	
	Vlaams Liga Tegen Kanker (VLK), Flemish League Against Cancer	Collaborator knowledge and policy
	Patienten Rat & Treff, German League for Patient’s Rights	Collaborator
	Belgian Cancer Foundation	Knowledge manager, PhD Social Sciences
	Cédric-Hèle Institute, Flemish Institute for Psycho-oncology	Collaborator

In total, three rounds of discussion took place, and each lasted 3 h on average. The first session aimed at clearly defining the issue(s) to be addressed by the literature review, the target group and the timeframe and at identifying additional experts or stakeholders to be invited. The second session took place 4 months later and focused on the presentation of the first results of the literature review and on the results from unpublished national studies. In this session, experts and stakeholders were asked the following questions: “Do these results fit with your experience and expertise? To which extent do they apply in the Belgian context (what is their strength)? Which features from the healthcare or employment system would you add as impeding or facilitating factors?”

The third session was dedicated to the final validation, combining the results from the literature, balanced and refined according to the Belgian social and economic context, and expert’s input.

After each session, the content of the discussion was reported ad verbatim by the coordinator and sent to all participants who were given time to complete or refine their statements. The final versions of the reports, available in French and Dutch, were used to draft this paper.

## Results

### Included studies

After merging the studies found in the three retained systematic reviews [9–11], and the database search, after eliminating duplications, 43 studies were included in the analysis. Nine risk factors were identified; these are presented according to the international classification by Collins et al. in 2013 [16]. The main results are presented in Table 3, and the Additional file 1 contains a detailed presentation of the included studies.

**Table 3:** Summary of results from the literature: risk factors, relevance of studies and main results

Factors	*n* studies	*n* studies with significant results	Relevance			Results
			High (low risk of bias)	Moderate (unclear risk of bias)	Low (high risk of bias)	
Socio-demographic						
Age	19	12	4	4	4	Women aged ≥50 and men aged ≥55 are more at risk.
Education or income	15	9	3	3	3	Low educational and income levels predict prolonged time to RTW.
Disease and treatment-related						
Cancer site	13	11	3	4	4	Head and neck, lung and breast cancers and leukaemia impede RTW.
Stage	7	6	1	3	2	Advanced cancer stages substantially lengthen sickness leave.
Treatment	21	18	1	9	8	Chemotherapy and combination of therapies are negatively associated with RTW.
Symptoms	11	11	0	6	5	Fatigue, pain and depression are the main impeding symptoms.
Work-related						
Type, sector and job demands	13	12	4	4	4	Lower occupational class, private sector and demanding jobs impede the (time to) RTW.
Employers’ and colleagues’ support	7	7	0	0	7	Support of colleagues and employers predict quicker and easier RTW.
Personal and subjective						
Value of work	7	7	1	1	5	The (re)evaluation of the importance of paid work substantially affects the choice to RTW.
Total	43		10	19	14	

### Socio-demographic factors

#### Age

Nineteen out of the 43 studies addressed age, 12 of which with significant results (*p* ≤ 0.05). Results from the 12 studies were consistent and showed that in both female and male cancer survivors, aged over 50 and 55 years, respectively, there was an increased risk for delayed RTW or early retirement. In one study [17], a higher risk not only for employed cancer survivors over 50 years but also for those under 29 years was reported. In another study [18], it was reported that older cancer survivors were more inclined to decide—deliberately—not to return to work.

#### Education and income

Since educational level and income are two related socio-demographic aspects, in analysis, the two factors were treated as one. Fifteen out of the 43 included studies considered the educational level and/or income, of which nine obtained significant results (*p* ≤ 0.05). Studies revealed that a high educational (university) level and a high income predict a shorter time to RTW. This association seemed particularly true for men. One of the studies [19] reported a same trend in the general population, irrespective the reason of sick leave.

### Disease-related factors

#### Cancer site

Thirteen out of the 43 included studies investigated the impact of the cancer site on the employment outcomes of cancer survivors, of which 11 had significant results (*p* ≤ 0.05). Results of the 11 studies converged and showed that cancer survivors with stomach, thyroid and skin cancers have higher employment rates compared to lung cancer, leukaemia and CNS cancer survivors. Others reported that kidney, bladder and thyroid gland cancer survivors had similar outcomes regarding time to job loss compared to disease-free individuals. In the studies also, a higher median time to RTW in breast cancer survivors was reported.

#### Stage

Seven out of the 43 included studies dealt with the impact of the disease stage on the employment outcomes (being reported in stage or by the size of the tumour). Six of them had significant results (*p* ≤ 0.05) regarding the association between stage and employment outcomes. In the case of advanced stages of the disease, all studies reported both a prolonged time to RTW and more disabilities that impeded the process of resuming work.

#### Type of treatment

Twenty-one out of the 43 included studies reported on the association between treatment modalities and employment status of cancer survivors, of which 18 presented significant results (*p* ≤ 0.05). Of these, studies on breast cancer survivors reported differences in sick leave. Women who had a mastectomy or chemotherapy had a prolonged absence compared to women who underwent breast-conserving surgery.

Other studies reported that cancer survivors who had chemotherapy or a combination of therapies (e.g. surgery, radiotherapy and chemotherapy) had a fourfold increased risk of not resuming work in the first (or even the three) following year(s) after treatment, compared to cancer survivors who had only surgery or one type of treatment. The responsibility of reduced cognitive ability caused by chemotherapy has been emphasized in all studies having questioned patients who had received chemotherapy. Also, in studies on the effects of chemotherapy, the association of reduced cognitive ability and chemotherapy treatment was reported.

#### Symptoms

Eleven out of the 43 included studies addressed the impact of symptoms on the ability of cancer survivors to resume or maintain work. Only one presented quantitative results [20]. Six reported fatigue as the main factor impeding RTW of cancer survivors; other relevant symptoms reported were pain and distress. Seven focused on a specific cancer site, which implies that besides the most common symptoms like fatigue, distress and pain, other symptoms exist, such as dry mouth, incontinence and lymphedema. These are also important symptoms that seem to relate to a specific cancer site. Another important recurring symptom reported was cognitive impairment [21]. It may lead to difficulties in concentration and memorization and may cause emotional strains, due to the loss of self-confidence or confidence from colleagues.

### Work-related factors

#### Type, sector and job demands

Thirteen of the 43 included studies explored one or several aspects related to the type of job, sector of activity or job demands, 12 of which reported significant results. Several job characteristics were reported to influence the time to RTW of cancer survivors.

Studies reported that manual work, self-employment and working in the private sector were factors that negatively affect the RTW of cancer survivors. One study [22] on cancer survivors and RTW reported a reduction in the number of working hours still visible 6 years after diagnosis and treatment.

Studies also revealed that the workload, as assessed by cancer survivors, was an important factor that negatively affected their RTW.

#### Employers’ and colleagues’ support

Seven out of the 43 included studies explored the support provided by colleagues and employers to cancer survivors. In the quality assessment, all seven studies scored a low relevance, probably because of their study design, as they all collected data from a small group of respondents. Despite the weakness of the study designs, we observed some of the results to concur. That is, keeping in touch with colleagues at work helped cancer survivors to RTW (even partially) quicker. It also allowed a better understanding of colleagues regarding the cancer survivors’ limitations and helped them to tailor their work adequately as to fit their capacities to job demands [23]. Opposite to this, little or a total absence of communication between the cancer survivor and colleagues and/or employers (the direct supervisor or human resources) tends to be a factor that prolongs time to RTW and negatively influences the quality or adequacy of the RTW [24].

### Personal and subjective factors

#### The (new) meaning of work

Seven out of the 43 included studies examined the meaning of work for cancer survivors.

Of these, the one with a high relevance (a survey of 646 women with breast cancer) [25] reported that a majority of respondents gave less meaning to work, once diagnosed and treated. In addition, 86 % of those who stopped working did it on their own initiative.

The one study with moderate relevance [26] observed two opposite opinions on the meaning of work for cancer survivors. Those who resumed work did it mainly because they considered that work was bringing contentment and structure to everyday life, while those who chose not to RTW did it mainly because they considered themselves as sick and, therefore, not able to work.

The five studies with a low relevance confirmed these tendencies and reported that the most impeding factors of RTW were symptoms, e.g. especially fatigue and pain, and the changed relations with colleagues.

### Results from the consultation with experts

Each of the nine risk factors identified in the literature review was presented and discussed with the experts. In addition to these nine factors, experts added three main aspects to be considered in facilitating the RTW of cancer survivors: (1) the role of health professionals in the screening of cancer survivors at risk and their early sensitization to consider the existing options for RTW and to consult with their GP, social security physician and occupational physician; (2) a thorough and systematic evaluation of the (remaining) work capacities; and (3) revised employment policies that promote maintaining and hiring cancer survivors with prolonged sickness absence. These results are described hereafter.

Based on the results of the literature review and from national unpublished work, the experts divided the four main categories of factors into two even groups, i.e. non-modifiable factors and factors open to positive change that may enhance RTW of cancer survivors.

Regarding socio-demographic factors, experts acknowledged that cancer survivors aged over 50 years and less than 35 years had the highest risk of facing difficulties in employment. For the younger ones, difficulties mainly are related to their lack of experience combined with the seriousness of their disease. Also, reluctance of employers to keep them on board or to hire them, fearing relapse and lower productivity, may play a part. For the elderly, difficulties are mostly due to comorbidities, the burden of symptoms and side effects of treatment.

Related to education, experts confirmed, as found in the literature, that cancer survivors with a low level of education often have physically demanding jobs and that the level of their income (usually low) does not present an important incentive to resume work, as to preserve a high standard of living.

Regarding cancer site and stage, experts emphasized the importance to assess these together to draw conclusions on their impact on work ability. These two factors assessed together were believed to provide more accurate information related to the physical and emotional burden of the disease.

The group of experts reported that in the Belgian context, it is important to take into account the region the patient lives in, as employment policy is a regional prerogative. The most remarkable and unique example comes from the Province of Limburg, where employers have been sensitized to physical, cognitive and emotional difficulties of cancer survivors and have been invited to sign a charter committing their company to better support the employed cancer survivors and to positively discriminate others by hiring them.

Regarding the work-related factors, the consulted experts reported the need for more effort to adapt the workload (working time, work schedule, business trips, etc.) or the workstation to the capacity of survivors or to their treatment and recovery needs. In parallel, cancer survivors also need to be better prepared, physically and emotionally, offering them more medical and paramedical support.

Experts emphasized that besides the physiological aspects, the workplace (e.g. the direct supervisor and colleagues) also needs to be prepared for the RTW of cancer survivors who can present temporarily limited capacities or emotional distress. Experts reported it is best to avoid a long period of rupture with the social and professional environment. That is, the length of the absence has a negative impact on the quality of the RTW, making it harder for cancer survivors who were alienated from the workplace to RTW.

Small-sized companies and the private sector have been presented in the literature as risk factors for RTW of cancer survivors. However, experts have reported that national investigations and surveys have shown the contrary [27]. Experts stated that a significant number of small- and medium-sized enterprises (SMEs) in Belgium are particularly willing to adapt the workstation or schedule in order to keep cancer survivors with specific experience and knowledge and who, e.g. have a longstanding relationship with clients. Again, this particular insight serves the experienced (often older) cancer survivors but does not present an advantage for the younger ones, unless they are very qualified (i.e. those with a high educational level).

Related to the more personal and subjective factors, though they are difficult to assess and modify, experts reported that in cancer survivors, addressing RTW issues at an early stage may positively influence the evaluation of the meaning of work. To simply address the work issue with cancer survivors just before the end of their (curative) treatment seems to already raise awareness and questions, for which they can be referred to social security advisers. Also, in case of good prognosis, the reassurance about their future abilities to resume their daily activities(albeit into different extents), can facilitate the RTW process.

## Discussion

This rapid review aimed to collect information in order to provide recommendations to the Belgian government as to facilitate RTW of cancer survivors. Results were validated, rated and put into the Belgian context. Nine risk factors that seem to hinder RTW of cancer survivors were identified. In discussing the literature, the consultation of experts resulted into the categorization of the risk factors into two groups, i.e. those being modifiable, offering room for enhancement of RTW of cancer survivors, and those which are not. Next, based on expert consultation, we identified features of the healthcare, social security and employment systems that can be adjusted to minimize the (negative) influence of risk factors on RTW of cancer survivors.

Most socio-demographic and health-related factors are non-modifiable, but their assessment is important, since it allows the identification of those workers who would be at risk, leading to the early planning of adequate rehabilitation plans, tailored to the physiological or psychosocial needs of the worker.

The majority of studies that included breast cancer survivors reported very poor outcomes, at all stages. This brings us to the hypothesis that beyond site and stage, gender could be a risk factor. None of the studies included a report on gender differences. But we know from the literature that significant differences related to sickness absence between genders exist, presenting more and longer sickness absence among women, irrespective of cancer site [28]. Further research on the association of gender and RTW of cancer survivors needs to be performed to confirm or reject this hypothesis.

The work-related, subjective and personal factors can be modified to ensure the ability and readiness of (ex) cancer patients to resume work. Indeed, the emotional and physical workload may include aspects that can be modified, temporarily or permanently. These modifications could be managed and coordinated by different health professionals, in agreement with the employer, the line manager, direct supervisor or the human resources service. The number of involved actors actually contributes to the lack of clarity on their prerogatives [29], and at the moment, nobody takes the responsibility.

The group of experts emphasized two more important aspects: first, the role of the *intra-mural* healthcare professionals in the early screening and referral to occupational supportive care. Albeit the identification of cancer survivors at risk is possible, there is a need to assess the feasibility and acceptability of such a role and to foresee adequate tools and training for those involved.

The second important aspect concerns the responsibility of employers, medical advisors and occupational physicians in maintaining or hiring (ex) cancer survivors at the workplace. A better and systematic evaluation of the *remaining* capacities of cancer survivors can lead to tailor-made rehabilitation and reintegration plan.

At the moment in Belgium, as in Spain and in Sweden, employers cover 1 month of sick leave pay, contrary to the Netherlands where 24 months are covered [30]. The financial responsibility of employers combined with the inability to terminate the contract may present an added value in the vocational rehabilitation of cancer survivors and reduce the time to RTW.

## Conclusions

This rapid review and the insights from experts enabled the identification of a list of factors impeding the RTW of cancer survivors. The awareness of health professionals with regard to modifiable factors leads to early identification of survivors at risk and may support them better.

From the healthcare system perspective, the list of risk factors that we drew (Table 3) can be used by both health professionals, to systematically identify cancer survivors who need more care and support, and health and social security policy-makers, to adapt the legislation making it more supportive for high-risk groups, such as the young-aged and blue collar workers.

An improved communication between physicians is required to ensure tailored multidisciplinary care. Training to use existing and validated tools for assessing work capacities should be offered to health professionals and to all those actors involved in the RTW process.

At the moment in Belgium, no consensus clearly exists either on the *reasonable* adaptation of the work or the role of actors. Social security and employment policies should support both employers and survivors. The Internal Services for Prevention and Protection at Work or the unions could develop recommendations on how to prepare and inform the direct supervisor and colleagues.

### Limitations and needs for future research

The choice not to focus on randomized control trial studies made it impossible to quantify the strength of the identified factors. Nor was it possible to prioritize the factors according to their relevance related to the professional reintegration process of cancer survivors. We encourage future cohort studies to quantify, compare and demonstrate the impact of the risk factors.

Unlike systematic reviews, at the moment, no quality insurance checklist exists regarding the rapid review process. The development of a PRISMA checklist for rapid reviews, taking into account expert opinions, could increase the quality and comparability of such studies.

## Additional files

Additional file 1:
**List of included studies.** (DOCX 28 kb)

## Footnotes

1 Five-year prevalence, reported by the GLOBOCAN 2012 (IARC).
