# Supplementary material for: Barriers and opportunities for return-to-work of cancer survivors: time for action—rapid review and expert consultation
Source: Syst Rev. 2016 Feb 24;5:35. doi: 10.1186/s13643-016-0210-z (PMC4765094; doi:10.1186/s13643-016-0210-z)
Supplement: Additional file 1: — List of included studies. (DOCX 28 kb) [file 13643_2016_210_MOESM1_ESM.docx]

# Appendix. List of included studies

| **Studies** | **n patients**  **n control** | **Focus** | **Study design** | **Cancer site** | **Factors addressed** | **Points /29** |
| --- | --- | --- | --- | --- | --- | --- |
| Amir et al.  2008 UK | 41 patients  No control | To describe the experience to RTW following cancer diagnosis and treatment | Retrospective qualitative study | 17 sites | Medical/employer’s support; value of work | 15 |
| Bains et al.  2011 UK | 49 patients  No control | To examine how treatment/symptom has an impact on perceptions of work ability and work decisions | Longitudinal cohort study | Colorectal | Value of work; self-assessment; treatment | 15 |
| Balak et al.  2008 Netherlands | 72 patients  No control | What is the RTW rate following early-stage breast cancer? What is the effect of the type of treatment and cancer-related symptoms on RTW? | Prospective cohort study | Breast | Disease and treatment; | 21 |
| Bennett et al.  2009 New Zealand | 68 patients  No control | To describe changes in employment and household income | Cross sectional qualitative study | All | Socio-demographic; disease/treatment; employer’s support | 20 |
| Borget et al.  2007 France | 194 patients  No control | To compare the frequency/duration/cost of sick leave for follow-up control between rhTSH and withdrawal | Prospective and Retrospective study | Thyroid | Treatment | 20 |
| Bouknight et al.  2006 USA | 416 patients  No control | To identify correlates of RTW in employed breast cancer survivors | Prospective qualitative study | Breast | Work; employer’s support | 21 |
| Boykoff et al.  2009 USA | 74 patients  No control | To document the impact of chemotherapy on the social and professional life | Cross sectional qualitative study | Breast | Treatment | 14 |
| Bradley et al.  2005 USA (a) | 267 patients  283 controls  256 controls | To explore employment outcomes 6 and 12  months after diagnosis | Prospective cohort study | Prostate | Treatment | 23.5 |
| Bradley et al.  2005 USA (b) | 445 patients  372 controls | To examine the consequences of breast cancer for women’s labor market attachment at the 6-month period following diagnosis | Longitudinal study | Breast | Probability of employment; education; age; | 20 |
| Bradley et al.  2006 USA (c) | 239 breast  206 prostate  No control | To examine the number of days employed patients undergoing treatment for either breast or prostate cancer were absent from their jobs | Retrospective cohort study | Breast - prostate | Treatment | 20 |
| Buckwalter et al.  2007 USA | 666 patients  No control | To evaluate patients’ reported reasons for discontinuing employment following treatment | Prospective observational study | Head and neck | Disease and treatment | 19 |
| Carlsen et al.  2008 Denmark | 40.884 patients  196.109 controls | To investigate whether cancer survivors are at risk for unemployment after cancer | Longitudinal cohort study | 13 sites | Socio-demographic; work | 28 |
| Choi et al.  2007 Korea | 305 patients  No control | To investigate the impact of a cancer diagnosis on employment status, and to identify relevant associated factors | Prospective cohort study | Stomach, liver, colorectal  (only men) | Disease; treatment; work; socio-demographic | 15 |
| Cooper et al.  2013 UK | 290 patients  No control | To examine the role of clinical, socio-demographic, work and psychological factors in RTW | Prospective cohort study | Breast-Gynaeco- Head and neck- Urogenital | Self-assessment/perception; work | 21 |
| de Boer et al.  2008 Netherlands | 195 patients  No control | To assess the extent to which self-assessed work ability during treatment can predict RTW | Prospective cohort study | Breast; female genitals and uro | Self-assessment; socio-demographic | 16 |
| Eaker et al.  2011 Sweden | 4761 patients  23.805 controls | To examine the influence of a cancer diagnosis on work, marital status, sickness absence and income | Retrospective population-based cohort study | Breast | Disease; treatment; socio-demographic | 26 |
| Farley Short et al. 2004 USA | 1763 patients  No control | To study employment/disability/self-reported effects on employment | Retrospective qualitative study | All | Disease; self-assessment | 22 |
| Gudbergsson et al. 2006 Norway | 417 patients  417 controls | To explore job strain in Norwegian primary-treated cancer survivors compared to matched controls in the general population | Cross-sectional case-control study | Breast, testicular, prostate | Socio-demographic; | 26 |
| Grunfeld et al.  2012 UK | 55 patients  No control | To explore the experience of women who RTW | Longitudinal qualitative study | Gynaecological | Value of work; treatment | 14 |
| Johnsson et al.  2007 Sweden | 222 patients  50 controls | To investigate whether socio-economic and treatment-related factors were associated with problems in RTW | Randomized trial | Breast | Treatment; socio-demographic; disease | 22 |
| Johnsson et al.  2009 Sweden | 97 patients  No control | To assess factors that predict the RTW of women with early stage cancer | Prospective cohort study | Breast | Work; treatment | 17 |
| Johnsson et al.  2010 Sweden | 16 patients  No control | To identify factors of a successful return to the labour market from women’s perspective | Retrospective qualitative study | Breast | Treatment; value of work | 15 |
| Kennedy et al.  2007 UK | 29 patients  No control | To explore the factors that inﬂuence decisions about RTW either during or after cancer treatment | Qualitative exploratory study | Breast | Value of work; employer’s support; treatment | 14 |
| Kirchhoff et al.  2010 USA | 197 patients  No control | To investigate whether demographic/medical/ functional factors predict full-time work return | Prospective cohort study | Hematologic malignancy | Socio-demographic; disease; treatment | 18 |
| Lauzier et al.  2008 Canada | 459 patients  No control | To evaluate wage losses over the first 12 months after diagnosis | Prospective cohort study | Breast | Education; distance from hospital; treatment; financial aspects | 24 |
| Lee et al.  2008 Korea | 408 patients  994 controls | To compare employment status and work-related difficulties between stomach cancer patients and the general population | Cross-sectional case-control study | Stomach | Socio-demographic; treatment | 15,5 |
| Main et al.  2005 USA | 28 patients  No control | To describe the work of cancer survivors and to explore factors inﬂuencing decisions about work | Cross sectional qualitative study | 11 sites | Value of work; treatment | 17 |
| Maunsell et al.  2004 Canada | 646 patients  890 controls | To assess negative or involuntary changes in employment situation | Retrospective qualitative study | Breast | Value of work; self-assessment | 25 |
| Mols et al.  2012 Netherlands | 2892 patients  No control | To examine the socio-economic implications (work changes) of cancer survivorship | Retrospective qualitative study | Colorectal; melanoma; lymphoma; myeloma | Work | 21 |
| Moran et al.  2011 USA | 673 patients  4141 controls | To estimate the average effect of treatment on employment and working hours | Cross sectional case-control study | 13 sites | Work; | 26 |
| Munir et al.  2010 UK | 13 patients  No control | To investigate women’s awareness and perception of chemotherapy-induced changes and RTW decisions | Cross sectional qualitative study | Breast | Treatment; self-assessment; work | 14 |
| Noeres et al.  2013 Germany | 227 patients  647 controls | 1) Does breast cancer lead to an increased drop-out of paid work?; 2) do other factors explain breast cancer survivors retirement from work? | Prospective case-control study  Prospective/ two times cross-sectional? | Breast | Work; treatment; disease; socio-demographic | 23 |
| Park et al.  2008 Korea | 1398 patients  No control | To investigate the impact of cancer diagnosis on job loss and re-employment | Prospective quantitative cohort study | 21 sites | Socio-demographic; work; disease; treatment | 26 |
| Park et al. 2009  Korea | 4991 patients  12468 controls | To investigate whether cancer diagnosis effects employment status | Prospective case-control study | 14 sites | Socio-demographic; disease | 29 |
| Rasmussen et al.  2008 Denmark | 23 patients  No control | To analyse the meaning of work and working life for cancer survivors over time | Propsective qualitative study | 15 sites | Value of work; treatment | 18 |
| Roelen et al.  2011 (a)Netherlands | 5074 patients  No control | To analyze the RTW (sickness absence) after cancer | Prospective quantitative study | 6 sites | Disease; socio-demographic | 25 |
| Roelen et al. 2011 (b)  Netherlands | 3357 patients  No control | To investigate the trends of RTW after breast cancer | Longitudinal study | Breast | Work; socio-demographic | 19 |
| Roelen et al. 2011 (c)  Netherlands | 5234 patients  No control | To investigate association of demographics and occupational factors | Prospective study (survival analysis) | All | Socio-demographic; work | 25 |
| Shewbridge et al. 2012 UK | 55 patients  No control | To identify personal or treatment-related factors that influence patients who continue to work when undergoing ambulatory chemotherapy | Cross sectional survey | Breast, colorectal; lymphoma | Disease; treatment; employer’s and colleague’s support; | 9 |
| Sjövall et al.2012 Sweden | 2738 patients  12.246 controls | To observe sickness absence before and after the cancer diagnosis | Case-control study | Colon, rectal, breast, prostate, lung | Disease | 21,5 |
| Spelten et al. 2003 Netherlands | 235 patients  No control | To assess the impact of fatigue and cancer-related symptoms on RTW in cancer survivors | Prospective inception cohort study | All | Treatment; socio-demographics; work | 22 |
| Sultan et al.  2006 USA | 537 patients  No control | To identify factors that predict RTW and resumption of unlimited physical activity following open radical retropubic prostatectomy | Prospective cohort study | Prostate | Socio-demographic; work | 15 |
| Tevaarwerk et al. 2013 USA | 530 patients  No control | To examine whether symptoms may be targets for intervention to improve work outcomes | Cross sectional qualitative study | Breast, lung, prostate, colon | Disease/treatment; work; socio-demographic | 22 |
